# Does the use of acellular dermal matrices (ADM) in women undergoing pre-pectoral implant-based breast reconstruction increase operative success versus non-use of ADM in the same setting? A systematic review protocol

**DOI:** 10.1186/s13643-024-02564-7

**Published:** 2024-06-07

**Authors:** H. Cook, D. Zargaran, S. P. Glynou, S. Hamilton, A. Mosahebi

**Affiliations:** 1 Plastic Surgery Department, Royal Free Hospital, Pond Street, London, NW3 2QG UK; 2 Division of Surgery and Interventional Science, University College London, London, UK; 3 Imperial College London School of Medicine, London, UK

**Keywords:** Breast cancer, Implant-based breast reconstruction, Pre-pectoral breast reconstruction, Acellular dermal matrix, Complications, Implant failure, Patient satisfaction

## Abstract

**Background:**

Breast cancer is the most common malignancy among women in the UK. Following mastectomy, reconstruction is now integral to the surgical management of breast cancer, of which implant-based reconstruction (IBBR) is the most common type. IBBR initially evolved from pre-pectoral to post-pectoral due to complications, but with developments in oncoplastic techniques and new implant technology, interest in pre-pectoral IBBR has increased.

Many surgeons use acellular dermal matrices (ADM); however, there is little evidence in literature as to whether this improves surgical outcomes in terms of complications, failure and patient satisfaction. This review aims to assess the available evidence as to whether there is a difference in surgical outcomes for breast reconstructions using ADM versus non-use of ADM.

**Methods:**

A database search will be performed using Ovid MEDLINE, Embase, Cochrane Central Register of Controlled Trials, Cochrane Database of Systematic Reviews and Clinicaltrials.org. The search timeframe will be 10 years.

Studies will be screened using inclusion and exclusion criteria and data extracted into a standardised spreadsheet. Risk of bias will be assessed. Screening, extraction and risk-of-bias assessments will be performed independently by two reviewers and discrepancies discussed and rectified. Data analysis and meta-analysis will be performed using Microsoft Excel and R software. Forest plots will be used for two-arm studies to calculate heterogeneity and *p*-value for overall effect.

**Discussion:**

With the renaissance of pre-pectoral IBBR, it is important that surgeons have adequate evidence available to assist operative decision-making. Assessing evidence in literature is important to help surgeons determine whether using ADM for IBBR is beneficial compared to non-use of ADM. This has potential impacts for patient complications, satisfaction and cost to healthcare trusts.

**Systematic review registration:**

PROSPERO 2023 CRD42023389072.

## Introduction

Breast cancer represented 30% of female cancers in the UK in 2019 — the most common malignancy diagnosed among women [1]. Mortality rates have fallen by 40% despite a 25% increase in incidence in the same period [1]. These changes are, in part, due to the introduction of the UK breast cancer screening programme at the end of last century [2].

Surgical management of breast cancer has evolved since early described mastectomies [3]. Reconstruction is now integral to the surgical management of breast cancer as it has been shown to reduce psychosocial morbidity and increase greater patient satisfaction [4]. Since the conception of silicone implants of the 1960s, implant-based breast reconstruction (IBBR) has gained popularity [5]. Immediate IBBR is currently the most prevalent reconstructive procedure performed in the UK [6].

Initially, IBBRs took place in the pre-pectoral plane, but following reports of capsular contracture, skin flap necrosis, infection and implant exposure, there was a shift towards subpectoral techniques in the 1970s [4]. Despite pectoralis muscular coverage leading to reduced implant exposure and improved cosmesis, subpectoral IBBR has been associated with increased postoperative pain, animation deformity and functional deficits [7].

With the evolution of oncoplastic techniques to conserve breast tissue, alongside new implant technology, pre-pectoral IBBR has gained renewed interest among surgeons [4, 8]. Many surgeons use acellular dermal matrices (ADM) in such procedures [9]. ADM is said to improve the cosmetic appearance of pre-pectoral breast reconstruction and provide more flexibility with reconstructive size [10]. Nonetheless, it has also been reported to increase the risk of infection, seroma and skin necrosis [11].

The United States Food and Drug Administration (FDA) issued a statement in 2021 reiterating that the FDA has not approved or cleared ADM for use in IBBR and highlighting the risks associated with its use [12]. Further safety concerns have been raised recently with the recall of SurgiMend, an ADM produced by Integra, due to high endotoxin levels causing post-operative fever [13].

Current evidence in literature regarding pre-pectoral ADM use for IBBR is limited, and there is scant comparison with non-ADM use in the same setting. Reporting of complications and patient quality of life is inconsistent. A systematic review was performed to explore surgical outcomes and quality of life for patients undergoing pre-pectoral IBBR with or without ADM.

## Methodology

This review has been registered with the International Prospective Register of Systematic Reviews (PROSPERO), part of the National Institute for Health Research (NIHR). Registration is as follows: PROSPERO 2023 CRD42023389072 [14].

### Study question

This study aims to compare operative success for patients undergoing pre-pectoral IBBR with or without the use of ADM, defined by post-operative complications, implant failure and patient quality of life.

### Literature search

A systematic literature search has been conducted with the assistance of the Royal College of Surgeons of England. Databases searched were Ovid MEDLINE, Embase and Cochrane Central Register of Controlled Trials (CENTRAL) and Cochrane Database of Systematic Reviews (CDSR). Clinicaltrial.org was also searched for ongoing studies. The search timeframe was 10 years given the increase in popularity of pre-pectoral IBBR over sub-pectoral in this time period [15]. Searches included the following terms in various combinations and forms:

- Acellular dermal matrix (ADM)
- Mammaplasty, breast implantation and breast reconstruction
- Mastectomy
- Breast cancer
- Post-operative complications and treatment outcomes
- Quality of life

### Study selection and data extraction

Studies will be independently evaluated according to PICO criteria (Table 1) and exclusion criteria (Table 2) by two review team members. Non-randomised (retrospective and prospective) studies and randomised control trials will be accepted. Systematic reviews will be accepted only if they do not contain references already included in search results. Titles and abstracts will be screened using exclusion criteria and then read in full. References of included studies will be screened for inclusion suitability. Information from studies that pass initial and full screening will be extracted by two reviewers and compared to mitigate for human error. Data will be collected in a standardised spreadsheet.

**Table 1:** Study Population, Intervention, Comparison and Outcomes (PICO)

Patient	1) Women undergoing pre-pectoral implant-based breast reconstruction with or without ADM 2) Women undergoing reconstruction for cancer treatment or prophylaxis 3) Immediate or delayed reconstruction 4) Unilateral or bilateral reconstruction
Intervention	Use of acellular dermal matrices (ADM) during breast reconstruction procedures
Comparison	Non-use of ADM during breast reconstruction procedures
Outcome	Operative success, defined by the following: 1) Complications 2) Failure 3) Patient quality of life

Outcome 1 — complications — will include short-term outcomes such as seroma, haematoma, wound breakdown, nipple necrosis, infection and long-term outcomes such as capsular contracture, rotation and rippling. Outcome 2 — failure — will be defined as complications resulting in implant removal or explantation. Outcome 3 — quality of life (QoL) — will encompass all QoL measuring tools used in included studies; follow-up should be for a minimum of 3 years

**Table 2:** Exclusion criteria

Exclusion criteria	• Secondary reconstructive procedures such as reconstruction revision • Aesthetic or cosmetic procedures • Sub-pectoral implant placement • Non-implant-based reconstruction, for example autologous free flaps • Animal or cadaveric studies • Systematic review including papers already present in results

Alongside outcome data, demographics such as age, gender, body mass index, diabetes and smoking status will be extracted. Surgical factors such as immediate or delayed reconstruction and use of neo-/adjuvant chemotherapy and radiotherapy will also be extracted.

### Study quality

Study quality and risk of bias will be assessed using the Cochrane ROB 2 score for randomised control trials, ROBINS-I tool for non-randomised studies and ‘Risk of Bias in Systematic Reviews Tool’ (ROBIS). Studies will be independently reviewed by two team members and scores correlated. Protocol amendments will be documented via updates on PROSPERO and reflected in the systematic review final manuscript.

### Statistical analysis

Analysis will be performed on Microsoft Excel and R software. Risk ratio with 95% confidence intervals will be calculated with combined data comparing ADM use versus non-ADM use. Forest plots will be created for two arm studies, including heterogeneity and *p*-value for overall effect. A *p*-value less than 0.05 will be considered significant. In heterogenous results, sub-group analysis will be performed. Publication bias will be assessed using funnel plots.

## Discussion

With the renaissance of pre-pectoral IBBR, it is important that surgeons have an adequate evidence base to enable operative planning in the patient’s best interest.

Arguably, pre-pectoral IBBR is beneficial both in the short term (reduced operative time and postoperative pain) and the long term (reduced risk of animation deformity and functional loss) [7]. The use of ADM has significantly contributed to increased pre-pectoral IBBR rates, alongside implant technology improvement and access to intra-operative perfusion assessment [4, 8].

The authors have found three prior literature reviews examining pre-pectoral IBBR. Wagner et al. appraise complication rates for all pre-pectoral IBBRs. The focus of the review is predominantly overall complication rate, although there is some comparison of ADM use versus non-ADM use [16]. Patient quality of life was not a focus of the review. Ching et al. assess patient quality of life for IBBR comparing subpectoral and pre-pectoral [7] — although pre-pectoral data was useful to assess, there was minimal comparison between ADM and non-ADM use as this was not the focus of the review. Lastly, Salibian et al. investigated pre-pectoral IBBR using only ADM/mesh and did not compare with non-ADM use [17]. Although all reviews contribute to the evidence base on pre-pectoral IBBR, none specifically compare ADM use vs non-ADM use. The authors feel there is a need for a systematic review and meta-analysis on this topic given the widespread use of ADM [9] and its cost to the health service [18]. Such information is important for surgeons making treatment decisions and to inform future research on the topic.

## Data Availability

Not applicable.

## Abbreviations

ADM: Acellular dermal matrix
IBBR: Implant-based breast reconstruction
PROSPERO: International Prospective Register of Systematic Reviews
NIHR: National Institute for Health Research
PICO: Population, Intervention, Comparison and Outcomes
ROBIS: Risk of Bias in Systematic Reviews Tool
